# Kidney stones and dietary intake in adults: a population-based study in southwest Iran

**DOI:** 10.1186/s12889-024-18393-1

**Published:** 2024-04-04

**Authors:** Bahman Cheraghian, Alipour Meysam, Seyed Jalal Hashemi, Seyed Ahmad Hosseini, Amal Saki Malehi, Dinyar Khazaeli, Zahra Rahimi

**Affiliations:** 1 Department of Biostatistics and Epidemiology, School of Public Health, Ahvaz Jundishapur University of Medical Sciences, Ahvaz, Iran; 2 Department of Nutrition, Shoushtar Faculty of Medical Sciences, Shoushtar, Iran; 3 Alimentary Tract Research Center, Clinical Sciences Research Institute, School of Medicine, Ahvaz Jundishapur University of Medical Sciences, Ahvaz, Iran; 4 Nutrition and Metabolic Diseases Research Center, Clinical Research Institute, Ahvaz Jundishapur University of Medical Sciences, Ahvaz, Iran; 5 Pain Research Center, Ahvaz Jundishapur University of Medical Sciences, Ahvaz, Iran; 6 Department of Urology, School of Medicine, Chronic Renal Failure Research Center Imam Khomeini Hospital, Ahvaz Jundishapur University of Medical Sciences, Ahvaz, Iran

**Keywords:** Kidney stones, Dietary intake, Population-based study, Iran

## Abstract

**Background:**

The prevalence of kidney stones is on the rise globally. Several risk factors, including lifestyle, contribute to the formation of kidney stones. Nevertheless, there is a contentious debate about the relationship between diet and kidney stones. Therefore, our study aimed to assess the relationship between macronutrients and micronutrients and the formation of kidney stones.

**Methods:**

This population-based cross-sectional study was conducted in the baseline phase of the Hoveyzeh Cohort Study, focusing on adults aged 35–70 in southwest Iran. The information on demographic characteristics, anthropometrics, kidney stone history, and food frequency was collected. Chi-square and t-tests were utilized to assess the relationship between categorical and numerical variables with kidney stones. The ANCOVA and logistic regression models were used to evaluate the relationships while controlling for confounding factors.

**Results:**

Among 10,009 participants, the overall prevalence of kidney stones was 18.77% (95% CI: 17.99–19.53). A higher intake of carbohydrates [OR = 1.02 (95% CI:1.002–1.03), *p* = 0.026] and copper [OR = 1.04 (95% CI:1.01–1.09), *p* = 0.025] were found to be associated with kidney stones. No associations were found between the other assessed macronutrients or micronutrients and kidney stones (*p*-tvalues > 0.05).

**Conclusion:**

Our study’s findings indicate a correlation between diet and the formation of kidney stones. However, the relationship between dietary factors and kidney stones is complex, and further research is needed.

## Background

Kidney stones are solid mineral deposits formed from dissolved minerals in the urine. They are mainly excreted through the urethra [1]. The prevalence of kidney stones ranges from 1 to 15% worldwide [2]. In Iran, it ranges from 1.9 to 5.7%, with a higher rate observed in the western provinces [3, 4]. The recurrence rate of kidney stones is high, with approximately 50% of patients experiencing a recurrence within 10 years [5]. The annual cost of treating kidney stones in the United States was $2.1 billion in 2000 and is projected to exceed $4 billion by 2030 [6]. The formation of kidney stones can be influenced by various factors, including demographic characteristics and environmental factors [7].

Previous studies have shown that dietary factors play a significant role in the development of kidney stones. High salt intake, low water intake, low consumption of dairy products, low intake of tea, consumption of oxalate-rich foods, and consumption of processed foods are all associated with an increased risk of kidney stones [8, 9]. On the other hand, consuming higher levels of potassium, magnesium, fiber, and vitamin B6 is considered to be a dietary inhibitor for calcium-containing stones [10]. A study conducted in Switzerland found that the probability of developing kidney stones was higher with increased consumption of cakes, biscuits, and soft drinks, and lower consumption of nuts and seeds, fresh cheese, teas, and alcoholic beverages, particularly wine [11]. Moreover, reduced consumption of vegetables, coffee, and alcoholic beverages increased the probability of developing kidney stones [12].

Given the high risk of kidney stone recurrence and the costs of the disease on the healthcare system and society, understanding the risk factors, including nutritional factors, is crucial for the design of prevention and treatment strategies. However, conflicting results have been obtained regarding the factors associated with kidney stone formation [1–3, 7, 13, 14]. Furthermore, there have been few studies in Iran that have examined the nutritional factors influencing kidney stones among adults in the context of a population-based study. Therefore, the present study investigated the associations between nutritional factors and kidney stone disease among adult residents of southwest Iran.

## Methods

### Design study and participants

This was a population-based cross-sectional study in Southwest Iran. A total of 10,009 individuals, aged 35–70 years, were recruited for the enrollment phase of the Hoveyzeh cohort study (HCS) from May 2016 to August 2018 and were assessed in this analysis [15]. The Hoveyzeh cohort study is one of the branches of the Prospective Epidemiological Research Studies in Iran (PERSIAN) [16] focused on non-communicable diseases. Informed consent was obtained from all individuals who wished to participate in the study. The inclusion criteria were individuals aged between 35 and 70 years, residing in the Hoveyzeh district, without severe mental disorders, ability to answer the questionnaires independently. We excluded participants with missing data on kidney stones and frequency of food questionnaire (FFQ), as well as those unwilling to participate in the study.

The Ethics Committee of Ahvaz Jundishapur University of Medical Sciences approved the study protocol (IR.AJUMS.REC.1398.279). This study was conducted in accordance with the Helsinki Declaration and its later amendments. On the day of registration, we obtained written informed consent.

### Covariates

Each participant completed the questionnaires, and well-trained interviewers conducted the anthropometric measurements. The variables in this analysis included age groups (35–39, 40–44, 45–49, 50–54, 55–59, 60–64, and ≥ 65 years), sex (male, female), residence type (urban and rural), and marital status (single, married, widowed, and divorced). The education levels are categorized as illiterate, primary school, secondary school, high school, diploma, and university. The physical activity was assessed using the International Physical Activity Questionnaire (IPAQ), which records participants’ self-reported daily activity over a 1-year period. The physical activity and metabolic equivalent (MET) scores were reported for a 24-hour task [17, 18], and then they were categorized into quartiles in our analysis. The validity of the International Physical Activity Questionnaire (IPAQ) in Iran has been evaluated by Moghaddam et al. [19]. Body mass index (BMI) is calculated by dividing a person’s weight in kilograms by their height in meters squared (kg/m2). A BMI below 18.5 is considered underweight, 18.5–24.9 is within the normal range, 25.0-29.9 is classified as overweight, and over 30 is considered obese. In our study, a smoker is defined as an individual who has smoked at least 100 cigarettes in their lifetime.

### Dietary assessment

A semi-quantitative food frequency questionnaire (FFQ) consisting of 130 food items was utilized to evaluate individuals’ typical food consumption. The validity and reproducibility of the questionnaire were evaluated by Eghtesad et al. [20]. All the questionnaires were completed by a trained nutrition expert during face-to-face interviews with the participants. The participants reported the frequency of their consumption of each item over the past year, indicating whether it was daily, weekly, monthly, or yearly. Portion sizes of each food were converted to grams using household measures [21].

### Kidney stone assessment

The participants self-reported a history of kidney stones if the disease had been previously diagnosed by a doctor. Furthermore, all medical documents, including ultrasounds, photographs, laboratory reports, and surgical records, have been reviewed and verified by the team’s physicians. All medical documentation, including ultrasound images, photographs, laboratory reports, and surgical-related documents, has been thoroughly reviewed and verified by the team’s doctors.

### Statistical analysis

The data was described by reporting the frequency and percentage of the categorical variables, as well as the mean and standard deviation of the quantitative variables. The chi-square test evaluated the relationship between categorical variables. The Kolmogorov–Smirnov test and the normal Q-Q plot were utilized to assess the normality hypothesis for all food groups. The independent t-test was utilized to compare continuous variables between the two groups. We investigated the independent associations of the assessed factors with kidney stones using ANCOVA and unconditional multiple logistic regression, while controlling for confounding factors. In the univariate analysis, the criterion for initially entering variables into multiple regression models was set at *P* < 0.25. All reported *p*-values were based on two-tailed tests and were considered significant at a level of 0.05. We performed data analysis using STATA software version 15.

## Results

A total of 10,009 individuals who participated in the enrollment phase of the Hoveyzeh cohort study were evaluated in this analysis. The mean age of the participants was 48.76 ± 9.21 years, and approximately 60% of them were women. The overall prevalence of kidney stones was 18.77% (95% CI: 17.99–19.53). The prevalence was significantly higher in men (24.20%) compared to women (15.10%) (*P* < 0.001). Furthermore, individuals in the 50 to 54 age group exhibited the highest prevalence rate (21.9%) of kidney stones (Table 1).

**Table 1:** Prevalence rates of Kidney stones by sex and age groups

Age group (years)	Total			Male			Female			*P*-value
	n	case	Prevalence (%) (95% CI)	n	case	Prevalence (%) (95% CI)	n	case	Prevalence (%) (95% CI)	
**35–39**	1912	324	16.90(15.30 to 18.70)	732	164	22.40(19.50 to 25.50)	1180	160	13.60 (11.70 to 15.60)	< 0.001^*^
**40–44**	2025	362	17.90(16.30 to 19.60)	794	179	22.50(19.70 to 25.50)	1231	183	14.90 (13.00 to 16.90)	< 0.001^*^
**45–49**	1797	346	19.30(17.50 to 21.10	704	166	23.60(20.60 to 26.80)	1093	180	16.50 (14.40 to 18.80)	< 0.001^*^
**50–54**	1482	306	20.60(18.60 to 22.80)	608	150	24.70(21.40 to 28.20)	874	156	17.80 (15.40 to 20.50)	< 0.001^*^
**55–59**	1281	235	18.30(16.30 to 20.50)	541	137	25.30(21.80 to 29.10)	740	98	13.20 (10.90 to 15.80)	< 0.001^*^
**60–64**	798	175	21.90(19.20 to 24.90)	361	115	31.90(27.20 to 36.80)	437	60	13.70 (10.70 to 17.20)	< 0.001^*^
**≥ 65**	714	129	18.10(15.40 to 21.00)	286	65	22.70(18.10 to 27.90)	428	64	15.00 (11.80 to 18.60)	0.010^*^
**Total**	**10,009**	**1877**	**18.75(17.99 to 19.53)**	**4026**	**976**	**24.20(22.90 to 25.60)**	**5983**	**901**	**15.10 (14.20 to 16.00)**	**< 0.001** ^*^

*P < 0.05 was considered a statistically significant level in the chi-square test

Table 2 compares demographic, socioeconomic, and lifestyle variables between individuals with and without kidney stones. The study revealed that individuals with kidney stones were significantly more likely to be male, reside in urban areas, have a higher wealth status, engage in less physical activity, and be smokers compared to those without kidney stones. However, the educational level and BMI were not statistically different between the two groups.

**Table 2:** Characteristics of the participants based on kidney stone disease

Variables		Kidney Stone		*P*-value
		Yes	No	
**Age group**	**35–39**	324(16.90)	1588(83.10)	0.024^*^
	**40–44**	362(17.90)	1663(82.10)	
	**45–49**	346(19.30)	1451(80.70)	
	**50–54**	306(20.60)	1176(79.40)	
	**55–59**	235(18.30)	1046(81.70)	
	**60–64**	175(21.90)	623(78.10)	
	**>=65**	129(18.10)	585(81.90)	
**Sex**	**Male**	976(24.20)	3050(75.80)	< 0.001^*^
	**Female**	901(15.10)	5082(84.90)	
**Educational levels**	**Illiterate**	1120(18.00)	5089(82.00)	0.166
	**Primary school**	322(19.30)	1343(80.70)	
	**Secondary school**	142(21.10)	531(78.90)	
	**High school diploma**	149(20.1)	592(79.90)	
	**University**	144(20.00)	577(80.00)	
**Residence Type**	**Urban**	1243(20.10)	4933(79.90)	< 0.001^*^
	**Rural**	634(16.50)	3199(83.50)	
**Wealth index**	**Poorest**	324(16.20)	1676(83.80)	< 0.001^*^
	**Poor**	370(18.2)	1663(81.80)	
	**Moderate**	361(18.20)	1621(81.80)	
	**Rich**	402(19.90)	1621(80.10)	
	**Richest**	420(21.30)	1551(78.70)	
**BMI**	**Underweight**	23(15.40)	126(84.60)	0.535
	**Normal**	406(18.10)	1837(81.90)	
	**Overweight**	699(18.80)	3013(81.20)	
	**Obese**	749(19.20)	3156(80.80)	
**Physical activity (MET)**	**Q1**	522(20.90)	1981(79.10)	< 0.001^*^
	**Q2**	439(17.5)	2066(82.50)	
	**Q3**	421(16.80)	2087(83.20)	
	**Q4**	495(19.90)	1998(80.10)	
**Smoking**	**Yes**	472(22.60)	1617(77.40)	< 0.001^*^
	**No**	1405(17.70)	6515(82.30)	

*P < 0.05 was considered a statistically significant level in the chi-square test

After adjusting for age, sex, body mass index, physical activity, wealth index, and residence type, a significant difference in dairy product consumption between the two groups was observed using ANCOVA. The mean dairy consumption was significantly lower in the group with kidney stones (*p* = 0.014). Additionally, participants with kidney stones had a higher average intake of oils and sweets compared to those without kidney stones (*p* = 0.001). On the other hand, there was no significant difference in the average consumption of the other food items between individuals with and without kidney stones (*p* > 0.05). (Table 3).

**Table 3:** Comparison mean of food pyramid intake between two groups with and without kidney stones by the results of ANCOVA

Food pyramid groups	Kidney Stone	N	Mean^*^	SD	*P*-value**
**Meat&Beans**	No	8132	108.88	51.78	0.349
	Yes	1877	110.24	51.63	
**Fruits**	No	8132	410.69	253.74	0.109
	Yes	1877	399.11	244.97	
**Dairy**	No	8132	343.76	292.80	**0.014**
	Yes	1877	323.23	279.99	
**Vegetables**	No	8132	540.52	264.24	0.106
	Yes	1877	528.43	247.75	
**Grains**	No	8132	680.85	261.32	0.990
	Yes	1877	680.61	276.23	
**Oils & Sweets**	No	8132	130.07	83.64	**< 0.001**
	Yes	1877	139.13	94.45	

* Adjusted means after controlling for age, sex, body mass index, physical activity, wealth index, and residence type by ANCOVA

**P < 0.05 was considered a statistically significance level

To control for potential confounding variables, multiple logistic regression analysis was performed using two models. In Model 1, the macronutrients were assessed, while in Model 2, the micronutrients were assessed. According to the logistic regression analysis, the crude and adjusted odds ratios were calculated to evaluate the association between kidney stones and macronutrients. The results are presented in Table 4. After adjusting for potential confounders, an increased consumption of carbohydrates was associated with significantly higher odds of kidney stones [OR = 1.02 (95%CI:1.002–1.03), *p* = 0.026]. On the other hand, no significant associations were found between protein intake, fat intake, and fiber intake with kidney stones (all *p*-values > 0.05).

**Table 4:** Crude and adjusted Odds Ratios and their 95% confidence intervals for the assessed macronutrients using the Logistic Regression Model. (Model 1)

Variable	Crude Odds Ratio(95% CI)	*P*-value**	Adjusted Odds Ratio*(95% CI)	*P*-value**
Protein	1.002(1.001–1.004)	0.008	1.00(0.995–1.02)	0.983
Fat intake	0.99 (0.99–1.001)	0.593	-	
Fiber	1.008(1.003–1.012)	< 0.001	1.008(0.99–1.02)	0.088
Carbohydrate	1.004(1.002–1.007)	0.002	1.02(1.002–1.03)	**0.026**

* Adjusted after controlling for **Age Group, Sex, Educational level, Physical activity, BMI, and Wealth Status and Energy by** logistic regression

**P < 0.05 was considered a statistically significance level

In Model 2, after adjusting for potential confounders, among the evaluated micronutrients, the consumption of copper was found to significantly increase the odds of developing kidney stones. On average, for every 10 mg per day increase in copper intake, the odds of developing kidney stones increased by 4% [OR = 1.04 (95% CI:1.01–1.09), *p* = 0.025]. Although a higher intake of zinc slightly decreased the odds of developing kidney stones [OR = 0.95 (95% CI: 0.90-1.001) (*p* = 0.056)]. Moreover, no significant associations were found other assessed micronutrients with kidney stones (all *p*-values > 0.05). The details are presented in Table 5.

**Table 5:** Crude and adjusted Odds Ratios and their 95% confidence intervals for the assessed micronutrients using the Logistic Regression Model. (Model 2)

Variable		Crude Odds Ratio (95% CI)	*P*-value	Adjusted Odds Ratio (95% CI)	*P*-value
Calcium		1.002(0.990–1.015)	0.708	-	-
Iron		1.013(1.004–1.022)	**0.006**	0.97(0.95–1.03)	0.376
Magnesium		1.001(1.000-1.001)	**0.002**	0.99 (0.99–1.001)	0.210
Potassium		1.005(1.002–1.009)	**0.006**	1.00 (0.99–1.02)	0.954
Copper		1.121(1.047–1.201)	**0.001**	1.04 (1.01–1.09)	**0.025**
Zinc		1.018(1.004–1.032)	**0.010**	0.95(0.90-1.001)	0.056
Fluoride		1.004(1.001–1.007)	**0.003**	0.99 (0.99–1.01)	0.538
Manganese		1.048(1.022–1.074)	**< 0.001**	1.08 (0.96–1.22)	0.208
Vitamin B12		1.008(0.999–1.017)	0.093	1.08(0.96–1.22)	0.208
Vitamin E		1.009(0.997–1.022)	0.146	1.02(0.99–1.04)	0.123
Folate		1.05(1.02–1.09)	**0.003**	1.04(0.96–1.12)	0.309
Vitamin K		0.99(0.99–1.002)	0.459	-	
Vitamin A		1.003(0.99–1.02)	0.737	-	
Vitamin B6		1.003(0.999–1.006)	0.180	0.99(0.99–1.01)	0.791
Vitamin D		1.001(1.00-1.002)	**0.005**	1.001(0.99–1.002)	0.404

* Adjusted after controlling for Age Group, Sex, Educational level, Physical activity, BMI, and Wealth Status, Energy by logistic regression

**P < 0.05 was considered a statistically significance level

## Discussion

Our study aimed to investigate the relationship between food consumption and kidney stones. The main findings revealed a prevalence of kidney stones at 18.77%, with a higher prevalence among men, individuals residing in urban areas, those with high wealth status, less physical activity, and smokers. After adjusting for potential confounders, the participants with kidney stones had a lower average intake of dairy products and a higher average intake of oils and sweets compared to those without kidney stones. Furthermore, individuals with a higher carbohydrate intake had significantly higher odds of developing kidney stones.

We found a significant inverse relationship between kidney stones and dairy intake. In line with our results, one study found that consuming dairy is associated with a reduced risk of kidney stones [22]. However, increased milk intake is recommended for uric acid-related stones [23]. Therefore, policies that promote a diet high in dietary alkali load can help reduce the risk of kidney stone formation. These policies can support public health initiatives aimed at reducing the prevalence of kidney stones.

The results revealed a direct association between carbohydrate intake and kidney stones. In line with the results of our study, some research has suggested that consuming a high-carbohydrate diet may increase the risk of developing kidney stones [24]. High-carbohydrate diets may contribute to the formation of kidney stones by increasing calcium excretion in the urine. When the body breaks down carbohydrates, it produces an acid called oxalate, which can bind with calcium in the urine and form crystals. This, in turn, can lead to the formation of kidney stones. However, it is important to note that complex carbohydrates, such as those found in whole grains, fruits, and vegetables, are generally healthier than simple carbohydrates found in processed foods and sugary drinks [24, 25].

Our study revealed that there was no statistically significant association between kidney stones and vegetable consumption. In line with our study, the UK Biobank study showed that vegetable intake was not associated with the incidence of kidney stones [5]. However, several studies have indicated that consuming vegetables may reduce the risk of developing kidney stones [10, 26–28]. However, specific types of vegetables, especially leafy greens, have been identified as potential risk factors for the condition [29, 30]. This is because leafy greens contain a significant amount of oxalates, which can consequently increase the risk of developing oxalate-based stones, the most common type of kidney stone [12, 31]. The study conducted by Taylor and Curhan [32] also found no association between fruit consumption and kidney stones. Several studies have found that specific fruits, such as grapefruit, may elevate the risk of kidney stone formation. However, other fruits, such as oranges, have been found to have a protective effect against stone formation [33]. These contradictory results may be attributed to the varying levels of nitrates in different fruits.

We found that there was no statistically significant difference in the consumption of meat, beans, protein, fat, and fiber between the individuals with kidney stones and those without the condition. Previous prospective studies that have primarily focused on animal protein intake have yielded inconsistent evidence [34, 35]. These conflicting results may depend on the type of meat consumed and the balance of food types in the diet. A high-protein diet results in higher acid levels in the human body, leading to an increase in the excretion of calcium in the urine. Animal protein contains purines, which are broken down into uric acid in the body. Elevated levels of uric acid in the urine can also contribute to the formation of kidney stones [36]. However, these findings were inconsistent with the results of our study. A study showed that diets high in healthy fats, such as nuts, seeds, fish, and avocados, did not increase the risk of kidney stones [37]. However, other studies have shown a positive correlation between the consumption of fatty acids, such as arachidonic acid, and the excretion of urinary oxalate, which is a risk factor for the formation of kidney stones [38]. However, it appears that the type of fat intake can affect the risk of developing kidney stones. In contrast to our findings, some studies have suggested that a high-fiber diet may help reduce the risk of kidney stones [5, 35, 39]. Because these relationships are complex and not yet fully understood, policies that promote a balanced diet may reduce the risk of kidney stone formation.

Among the evaluated micronutrients, copper intake was directly associated with kidney stones. According to our study, research has shown that urinary copper levels were significantly higher in patients with kidney stones compared to healthy controls [40]. On the other hand, the results showed that intake of manganese, fluoride, zinc, iron, potassium, calcium, and magnesium was not associated with kidney stones. Although, one study showed that manganese deposition could be caused by the hemodialysis method itself [41]. Excessive fluoride may contribute to the development of calcium oxalate renal calculi [42]. Therefore, policymakers need to prioritize regulating fluoride levels in drinking water to prevent potential adverse effects on kidney health. On the other hand, a study reported increased odds of kidney stones with higher zinc intake [43]. The role of dietary potassium in the development of kidney stones is still unclear. A study found that potassium citrate may contribute to the formation of calcium-phosphate stones [44]. The magnesium level in a 24-hour urine analysis has also been found to be directly associated with oxalate levels, suggesting a role for magnesium in preventing stone formation by binding to oxalate [45]. It is important to note that while magnesium may be beneficial in preventing kidney stones, it is not a panacea. Some research, such as a prospective study on dietary calcium and other nutrients, found that a low-calcium diet was associated with an increased risk of kidney stone formation in a group of men. The study authors suggested that reducing calcium intake may lead to an increase in the absorption of oxalate, which can contribute to the formation of kidney stones [46]. It is important to maintain a balanced diet and consult with healthcare professionals for personalized recommendations regarding the intake of these micronutrients to minimize the risk of kidney stones, especially for individuals at high risk.

The results of our study revealed that there is no significant relationship between the consumption of vitamin K, A, B6, B12, and D and kidney stones. Vitamin K2, which is found in fermented foods and produced by bacteria in the intestine, regulates MGP and osteocalcin. These are important for proper mineralization and the inhibition of vascular calcification [47]. According to our study, research has shown that Vitamin B12 levels are not associated with kidney stones [48]. Additionally, a prospective analysis of over 193,000 participants found no statistically significant association between vitamin D intake and the risk of kidney stones [49]. There was no association between vitamin B6 intake and the occurrence of kidney stones in three large prospective cohorts [50, 51]. However, a study by Curhan et al. [52] demonstrated a negative correlation between vitamin B6 intake and the risk of stone formation in women.

This study has several limitations. First, it was a cross-sectional study, which means it cannot establish a cause-and-effect relationship. Second, our study relies on self-reported dietary data, which may be subject to recall bias. Third, we do not consider the composition of kidney stones, which can vary widely and may have different risk factors. On the other hand, the present study had several strengths. Our study had a large sample size, which can increase the statistical power and improve the precision of the estimates. Using a representative sample can improve the generalizability of the results. Additionally, we employed multivariate analysis to adjust for potential confounding factors, thereby enhancing the accuracy of the estimates and identify the independent effects of various risk factors.

## Conclusions

Our study’s results suggest a relationship between diet and the formation of kidney stones. It suggests that a high carbohydrate diet and high copper intake may increase the odds of kidney stones. Furthermore, dietary intake can reduce the odds of developing kidney stones. Further research is needed to better understand the complex interplay between genetics, diet, lifestyle, and the risk of kidney stones. It is recommended to seek advice from a healthcare professional before making substantial dietary changes or taking supplements.

## Data Availability

The datasets used and/or analyzed during the current study are available from the corresponding author upon reasonable request.

## References

[1] Sofia NHWT, Sanatorium T (2016). Prevalence and risk factors of kidney stone glob. J Res Anal.

[2] Romero V, Akpinar H, Assimos DG (2010). Kidney stones: a global picture of prevalence, incidence, and associated risk factors. Rev Urol.

[3] Rafiei H, Malekpoor F, Amiri M, Rahimi Madiseh M, Lalegani H (2014). Kidney stone development among older adults in Iran. J Indian Acad Geriatr.

[4] Nikpay S, Moradi K, Azami M, Babashahi M, Otaghi M, Borji M. Frequency of kidney stone different compositions in patients referred to a Lithotripsy Center in Ilam, West of Iran. J Pediatr Nephrol. 2016;4(3).

[5] Littlejohns TJ, Neal NL, Bradbury KE, Heers H, Allen NE, Turney BW (2020). Fluid intake and dietary factors and the risk of incident kidney stones in UK Biobank: a population-based prospective cohort study. Eur Urol Focus.

[6] Antonelli JA, Maalouf NM, Pearle MS, Lotan Y (2014). Use of the National Health and Nutrition Examination Survey to calculate the impact of obesity and diabetes on cost and prevalence of urolithiasis in 2030. Eur Urol.

[7] Adult S (2007). Urolithiasis in a population-based study in Iran: prevalence, incidence, and associated risk factors. Urol Res.

[8] Bhattacharya S, Joshi NK, Jain YK, Bajpai N, Bhardwaj P, Chaturvedi M et al. Dietary determinants of renal calculi: a case-control study from a Tertiary Care Hospital of Western Rajasthan. Cureus. 2022;14(11). 10.7759/cureus.31460 PMC9747953 36523708

[9] Dahl NK, Goldfarb DS. Nutritional prevention and treatment of urinary tract stones. Nutritional Management of Renal Disease: Elsevier; 2022. p. 685– 97.

[10] Legay C, Haeusermann T, Pasquier J, Chatelan A, Fuster DG, Dhayat N (2023). Differences in the Food Consumption between Kidney Stone Formers and nonformers in the Swiss kidney Stone Cohort. J Ren Nutr.

[11] AHMED L, Rebaz O (2023). The relationship between kidney stones and Dietary habits using XRF technique. J Phys Chem Funct Mater.

[12] Siener R (2021). Nutrition and kidney stone disease. Nutrients.

[13] Salmeh F, Yaghoubi T, Zakizadeh M, Yaghoubian M, Shahmohammadi S (2012). Evaluation of health behaviours in patients with kidney stones in Sari/Iran. Int J Urol Nurs.

[14] BASIRI A, SHAKH SN, Pakmanesh KHOUSHDELA, Radfar H. MH. Drinking water composition and incidence of urinary calculus introducing a new index. 2011. 21189428

[15] Cheraghian B, Hashemi SJ, Hosseini SA, Poustchi H, Rahimi Z, Sarvandian S (2020). Cohort profile: the Hoveyzeh Cohort Study (HCS): a prospective population-based study on non-communicable diseases in an arab community of Southwest Iran. Med J Islamic Repub Iran.

[16] Eghtesad S, Mohammadi Z, Shayanrad A, Faramarzi E, Joukar F, Hamzeh B (2017). The PERSIAN Cohort: providing the evidence needed for Healthcare Reform. Arch Iran Med.

[17] Aadahl M, Jørgensen T (2003). Validation of a new self-report instrument for measuring physical activity. Med Sci Sports Exerc.

[18] Ainsworth BE, Haskell WL, Whitt MC, Irwin ML, Swartz AM, Strath SJ (2000). Compendium of physical activities: an update of activity codes and MET intensities. Med Sci Sports Exerc.

[19] Moghaddam MB, Aghdam FB, Jafarabadi MA, Allahverdipour H, Nikookheslat SD, Safarpour S (2012). The Iranian version of International Physical Activity Questionnaire (IPAQ) in Iran: content and construct validity, factor structure, internal consistency and stability. World Appl Sci J.

[20] Eghtesad S, Hekmatdoost A, Faramarzi E, Homayounfar R, Sharafkhah M, Hakimi H et al. Validity and reproducibility of a food frequency questionnaire assessing food group intake in the PERSIAN Cohort Study. Front Nutr. 2023;10. 10.3389/fnut.2023.1059870 PMC10436288 37599697

[21] Shoja M, Borazjani F, Ahmadi Angali K, Hosseini SA, Hashemi SJ (2023). The dietary patterns derived by reduced-rank regression in association with Framingham risk score and lower DASH score in Hoveyzeh cohort study. Sci Rep.

[22] Taylor EN, Curhan GC (2013). Dietary calcium from dairy and nondairy sources, and risk of symptomatic kidney stones. J Urol.

[23] Brardi S, Verdacchi T, Paoletti G, Giovannelli V, Duranti E (2016). Does milk intake play a specific role in the pathogenesis of kidney stones?. Archives Ren Dis Manage.

[24] Taylor EN, Stampfer MJ, Curhan GC (2005). Diabetes mellitus and the risk of nephrolithiasis. Kidney Int.

[25] Siener R, Glatz S, Nicolay C, Hesse A (2004). The role of overweight and obesity in calcium oxalate stone formation. Obes Res.

[26] Meschi T, Nouvenne A, Ticinesi A, Prati B, Guerra A, Allegri F (2012). Dietary habits in women with recurrent idiopathic calcium nephrolithiasis. J Translational Med.

[27] Sorensen MD, Hsi RS, Chi T, Shara N, Wactawski-Wende J, Kahn AJ (2014). Dietary intake of fiber, fruit and vegetables decreases the risk of incident kidney stones in women: a women’s Health Initiative report. J Urol.

[28] Zhuo D, Li M, Cheng L, Zhang J, Huang H, Yao Y (2019). A study of diet and lifestyle and the risk of urolithiasis in 1,519 patients in Southern China. Med Sci Monitor: Int Med J Experimental Clin Res.

[29] Dai M, Zhao A, Liu A, You L, Wang P (2013). Dietary factors and risk of kidney stone: a case–control study in southern China. J Ren Nutr.

[30] De SK, Liu X, Monga M (2014). Changing trends in the American diet and the rising prevalence of kidney stones. Urology.

[31] Lieske JC, Rule AD, Krambeck AE, Williams JC, Bergstralh EJ, Mehta RA (2014). Stone composition as a function of age and sex. Clin J Am Soc Nephrology: CJASN.

[32] Taylor E, Curhan G (2008). Fructose consumption and the risk of kidney stones. Kidney Int.

[33] Barghouthy Y, Somani BK (2021). Role of citrus fruit juices in prevention of kidney stone disease (KSD): a narrative review. Nutrients.

[34] Turney BW, Appleby PN, Reynard JM, Noble JG, Key TJ, Allen NE (2014). Diet and risk of kidney stones in the Oxford cohort of the European prospective investigation into Cancer and Nutrition (EPIC). Eur J Epidemiol.

[35] Sorensen MD, Kahn AJ, Reiner AP, Tseng TY, Shikany JM, Wallace RB (2012). Impact of nutritional factors on incident kidney stone formation: a report from the WHI OS. J Urol.

[36] Ferraro PM, Taylor EN, Gambaro G, Curhan GC (2013). Soda and other beverages and the risk of kidney stones. Clin J Am Soc Nephrol.

[37] Park S, Pearle MS (2007). Pathophysiology and management of calcium stones. Urol Clin North Am.

[38] Haruo YN, Masai IM, Yamaguchi K (2002). Association of dietary fatty acids with urinary oxalate excretion in calcium oxalate stone-formers in their fourth decade. BJUI.

[39] Taylor EN, Fung TT, Curhan GC (2009). DASH-style diet associates with reduced risk for kidney stones. J Am Soc Nephrol.

[40] Atakan IH, Kaplan M, Seren G, Aktoz T, Gül H, Inci O (2007). Serum, urinary and stone zinc, iron, magnesium and copper levels in idiopathic calcium oxalate stone patients. Int Urol Nephrol.

[41] Esra A, Sultan O, Garip S, Ahmet, Ugur Y, Baki A (2018). The relation between brain MRI findings and blood manganese levels in renal transplantation, hemodialysis, and peritoneal dialysis patients. Int Urol Nephrol.

[42] Grases F, Perello J, Isern B, Costa-Bauza A (2008). The relationship between high fluoride intake and Nephrolithiasis. Curr Urol.

[43] Tang J, McFann K, Chonchol M (2012). Dietary zinc intake and kidney stone formation: evaluation of NHANES III. Am J Nephrol.

[44] Sudjai J, Vitoon P, Amorn P, Sirirat R, Piyaratana T, Kriang T, Suwantrai S, Chedsada N, Srinoi M, Pote. Sriboonlue. The effects of potassium and magnesium supplementations on urinary risk factors of renal stone patients. Journal of the Medical Association of Thailand Chotmaihet thangphaet, 87(3):255–263. Journal of the Medical Association of Thailand Chotmaihet thangphaet. 2004;87(3):255– 63. 15117041

[45] Sanaz T, Maryam T, Fatemeh T, Abbas B. Fahimeh, Bagheri, Amiri. Evaluating the associations between urinary excretion of magnesium and that of other components in calcium stone-forming patients. Int Urol Nephrol 2019:279–84. 10.1007/s11255-018-2036-1 30515733

[46] Curhan GC, Willett WC, Rimm EB, Stampfer MJ (1993). A prospective study of dietary calcium and other nutrients and the risk of symptomatic kidney stones. N Engl J Med.

[47] Rachel M, Holden., Sarah L, Booth (2007). Vascular calcification in chronic kidney disease: the role of vitamin K. Nat Rev Nephrol.

[48] Gearoid M, McMahon., Shih-Jen H, Rikki M, Paul T, Jacob FJ, Caroline SPM (2015). Fox. The association between vitamin B12, albuminuria and reduced kidney function: an observational cohort study. BMC Nephrol.

[49] Pietro M, Ferraro., Eric N, Taylor., Eric N, Taylor., Giovanni G, Gary C (2017). Curhan. Vitamin D intake and the risk of incident kidney stones the. J Urol.

[50] Pietro M, Ferraro., Eric N, Taylor., Eric N, Taylor., Giovanni G, Gary C, Curhan (2018). Vitamin B6 intake and the risk of incident kidney stones. Urol Res.

[51] Renato N, Pedro A, Ullah A, Jibril, Oyekunle B, Kamran, Hassan B, Joseph P, Idrissa S, Giovanna, Souza V, Alberto T (2020). Noor, Buchholz. Nutrients, vitamins, probiotics and herbal products: an update of their role in urolithogenesis. Urol Res.

[52] Gary C, Curhan., Walter C, Willett., Frank E, Speizer., Meir J. Stampfer. Intake of vitamins B6 and C and the risk of kidney stones in Women. J Am Soc Nephrol. 1999:840–5. 10.1681/ASN.V104840 10203369

